# Postural support requirements preferentially modulate late components of the gastrocnemius response to transcranial magnetic stimulation

**DOI:** 10.1007/s00221-022-06440-5

**Published:** 2022-08-25

**Authors:** Cassandra Russell, Nathan Difford, Alexander Stamenkovic, Paul Stapley, Darryl McAndrew, Caitlin Arpel, Colum MacKinnon, Jonathan Shemmell

**Affiliations:** 1 School of Medical, Indigenous and Health Sciences, Faculty of Science, Medicine and Health, University of Wollongong, Building 41, Northfields Avenue, Wollongong, NSW 2522 Australia; 2 Department of Neurology, University of Minnesota, Minneapolis, USA; 3 Department of Physical Therapy, College of Health Professions, Virgina Commonwealth University, Richmond, USA

**Keywords:** Posture, Balance control, Corticospinal, Cortico-reticulospinal, Brainstem, Reticular formation, Triceps surae

## Abstract

Mounting evidence suggests that motor evoked potentials (MEPs) recorded in upper limb muscles with postural support roles following transcranial magnetic stimulation receive contributions from both corticospinal and non-corticospinal descending pathways. We tested the hypothesis that neural structures responsible for regulating upright balance are involved in transmitting late portions of TMS-induced MEPs in a lower limb muscle. MEPs were recorded in the medial gastrocnemius muscles of each leg, while participants supported their upright posture in five postural conditions that required different levels of support from the target muscles. We observed that early and late portions of the MEP were modulated independently, with early MEP amplitude being reduced when high levels of postural support were required from a target muscle. Independent modulation of early and late MEPs by altered postural demand suggests largely separable transmission of each part of the MEP. The early component of the MEP is likely generated by fast-conducting corticospinal pathways, whereas the later component may be primarily transmitted along a polysynaptic cortico-reticulospinal pathway.

## Introduction

Seminal studies performed on macaque monkeys (Lawrence and Kuypers 1968a, b) helped to establish the roles of descending neural pathways. They determined the role of the corticospinal tract in voluntary movement and dexterity, and the role of ventromedial brainstem pathways, including the reticulospinal and vestibulospinal tracts in postural control, locomotion and movement integration. The corticospinal tract has been extensively studied due to its superficial location in the primary motor cortex, making it amenable to stimulation. However, considerably less is known about the organization and function of human brainstem pathways and their roles in the expression of motor impairment following lesions or disease of the nervous system. Transcranial magnetic stimulation (TMS) is a non-invasive stimulation technique that can be used to stimulate pathways descending from the primary motor cortex (Barker et al. 1985). Upon application, TMS can directly or indirectly induce action potentials in layer V pyramidal tract cells, which are propagated down the corticospinal tract to spinal motoneurons. This results in a motor evoked potential (MEP) in contralateral muscles that can be recorded with electromyography (EMG) (Barker et al. 1985). TMS-induced excitation has also been found to traverse corticoreticular synapses and activate the reticular formation (Fisher et al 2012).

By understanding if and when reticular formation excitation (and possibly excitation in other brainstem structures) contributes to TMS-induced MEPs that are transmitted along cortico-brainstem pathways, we may gain important insights into the role of motor pathways descending from the brainstem in posture and movement control.

**Fig. 1:**
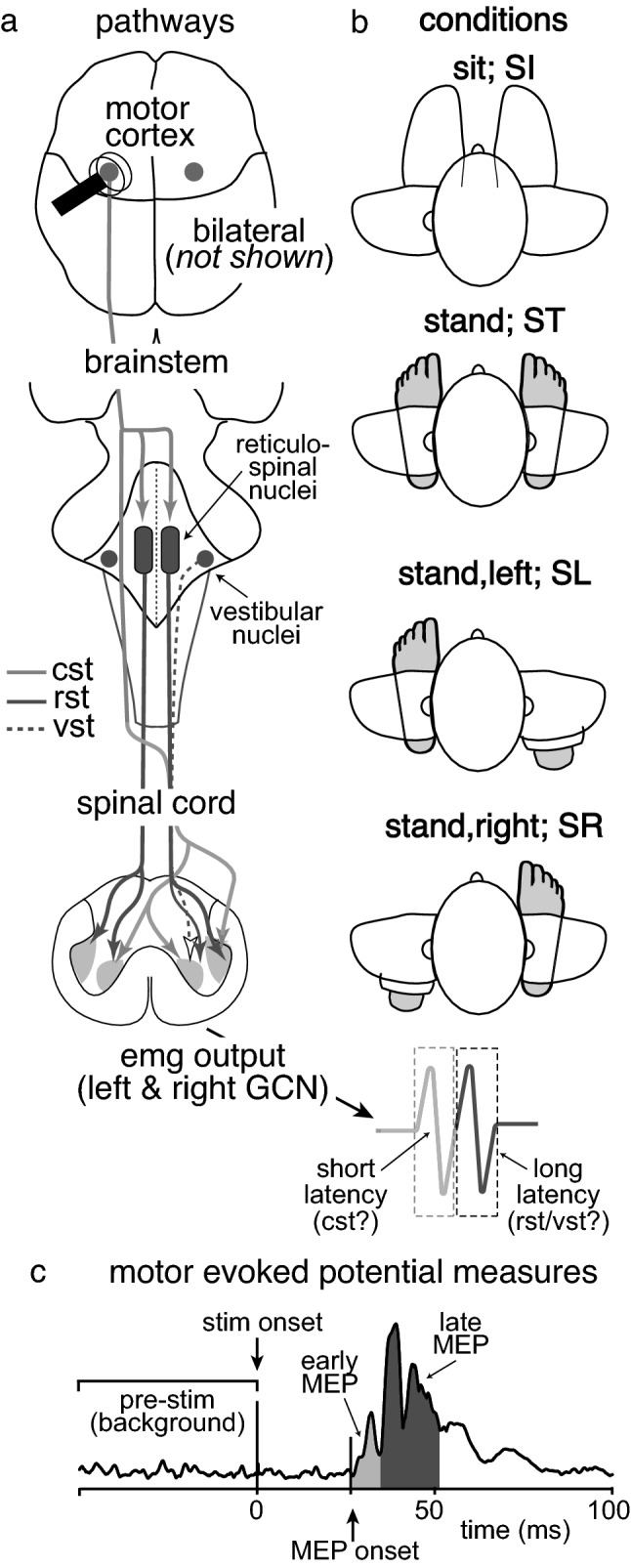
**A** Diagram showing the point of cortical stimulation and the descending motor pathways along which excitatory volleys may be transmitted following TMS. These include the corticospinal tract (cst), the reticulospinal tract (rst) and the vestibulospinal tract (vst). **B** Four major experimental conditions in which TMS was applied (excluding sham stimulation) with the foot providing postural support in each condition shaded. **C** Example of an MEP in the medial gastrocnemius following TMS (stim onset). Average EMG levels was calculated within a pre-stimulation window as well as the early (shaded light grey) and late (shaded dark grey) MEP

It has been well established since, that the earliest portions of TMS-induced MEPs are mediated by the fast-conducting corticospinal tract (Di Lazzaro and Ziemann 2013). Observations of late-arriving volleys in humans and reticular formation activation in monkeys, however, have provided evidence that later portions of the MEP receive contributions from a polysynaptic cortico-brainstem pathway, potentially involving the reticular formation or vestibular nuclei (Brum et al. 2016; Fisher et al. 2012) (see Fig. 1A). For example, MEPs in the pectoralis major ipsilateral to TMS (iMEPs) have been observed with onset latencies an average of 5.3 ms later than responses in the homologous contralateral muscle (MacKinnon et al. 2004). Other studies have found finger, wrist extensor and elbow flexor muscles to display iMEPS ~ 5.7 ms later than contralateral MEPs (Ziemann et al. 1999; Taga et al. 2021). Based on the longer latencies, the authors of these papers have suggested that iMEPs are transmitted along a slower or more complex pathway, putatively a cortico-reticulospinal pathway or cortico-propriospinal pathway. This idea is supported by observations that iMEPs are augmented following lesions of the contralateral corticospinal tract (Schwerin et al. 2008). An alternative explanation for longer latencies for iMEPs compared to MEPs in contralateral muscles is that the MEP onset is delayed and its duration modified by intracortical circuits, either within or between motor cortices in each cerebral hemisphere (Van Den Bos et al. 2017). Evidence that iMEPs can be elicited in a patient with complete agenesis of the corpus callosum (Ziemann et al. 1999), however, appears to make an interhemispheric pathway an unlikely substrate.

Evidence that individuals with damage to the corticospinal tract, either following stroke or due to hereditary spastic paraplegia (HSP), often display MEPs of longer onset latency and duration than neurologically intact individuals (Brum et al. 2016; Fisher et al. 2013), suggests that polysynaptic or slow conducting pathways may also play an important role in transmitting MEPs recorded in muscles contralateral to TMS. These observations in humans are supported by recordings from reticular formation cells in macaque monkeys, which demonstrate that reticular formation cells respond strongly to TMS of the primary motor cortex at a small delay after the stimulus (Fisher et al. 2012). Reticular formation neurons showed robust changes in firing rate from 1 to 25 ms after TMS, including short-latency excitatory responses consistent with activation of both direct and indirect cortico-reticular pathways. Based upon these results, and given differences in the length of cortico-brainstem fibres in the monkey and human, Fisher et al. (2012) suggested that the effects of reticular formation activation following TMS might be observed ~ 10 ms after MEP onset in human muscles. Despite this approximation, the contribution of brainstem input to the MEPs evoked distal or proximal muscles remains poorly defined.

Given the prolific use of TMS in research and clinical settings to estimate the state of the motor system, it is important to identify the neural circuits involved in transmitting excitatory and inhibitory signals to target muscles. In particular, estimates of ‘corticospinal excitability’ that rely on the amplitude of peaks of recorded muscle activity occurring in early and late portions of the MEP (e.g., measures of peak-to-peak MEP amplitude) may be subject to unnecessary variability if different descending pathways contribute to the MEP at different times. Given the potential for TMS to aid clinical decision making (e.g.,Smith and Stinear 2016; Lin et al. 2021), improving estimates of the physiological state of the descending motor pathways contributing to TMS-induced MEPs is an important goal. Due to the dominant role played by the brainstem reticulospinal and vestibulospinal systems in the control of upright posture (Lawrence and Kuypers 1968a, b; Lemon et al. 2012), descending commands transmitted along these pathways will be necessarily modulated according to the postural requirements of the task. In this study, we used this modulation to investigate the idea that early and late portions of TMS-induced MEPs are transmitted along separable descending pathways: the earliest parts of the MEP along direct corticomotoneuronal pathways and later portions of the MEP along subcortical pathways involving the brainstem. We aimed to determine whether early and late contralateral MEPs can be independently modulated by changes in the requirement for postural support by a target lower limb muscle. We hypothesized that postures in which the target muscle was responsible for providing anti-gravity support would preferentially increase the amplitude of late portions of TMS-induced MEPs (> 8 ms from MEP onset) relative to early (fast conducting corticospinal) components of the MEP. This would be due to the increased involvement of putative polysynaptic inputs from corticofugal (e.g., ventromedial brainstem) pathways (Di Lazzaro and Ziemann 2013). Specifically, we hypothesized that the amplitude of late components of the MEP would be greater in the right gastrocnemius during standing than during sitting and during right leg standing compared to left leg standing. Similarly, we hypothesized that late MEP amplitude in the left gastrocnemius would be greater during standing than during sitting, and during left leg standing compared to right leg standing. We predicted that if changes were observed in the amplitude of the early component of the MEP, smaller amplitudes would be observed during conditions in which the postural support provided by the target muscle was greatest, reflecting a reduced role for direct corticomotoneuronal fibres during the familiar balance tasks.

## Materials and methods

### Participants

All procedures and methodology used was approved by the University of Wollongong Human Research Ethics Committee (Approval No: 2018_075) and are consistent with the Declaration of Helsinki. Nine neurologically intact adults without musculoskeletal injury (4 female, 5 male, age 21–51 years) gave informed consent to participate in the study. Participants provided information regarding their routine physical activities and self-reported leg dominance.

### Electromyographic recordings

Surface EMG recordings were obtained bilaterally from the medial gastrocnemius muscles, chosen due to their important role in postural control during upright stance (Basmajian and De Luca 1964; Giulio et al. 2009) and their consistency of activation during pilot testing. The muscle bellies were prepared for EMG using SENIAM standard skin preparation guidelines (Hermens et al. 2000). A bipolar bar electrode (DELSYS Bagnoli, MA, USA; inter-electrode distance 10 mm) with conductive gel was attached to the skin surface with adhesive interface stickers (DELSYS Bagnoli sensor adhesive interface 2-slot, MA, USA). Tape was attached over each bar electrode to secure it. A ground electrode was placed on the right patella, following the same preparation process. The EMG signal was amplified (× 1000: Delseys Bagnoil-8 amplifier) and digitized by a Cambridge Electronic Design (CED) Power1401 data acquisition unit, before being recorded by Signal software (CED Signal v7, Cambridge, UK) at 20 kHz; this frequency was selected for its ability to capture the short output signal from the TMS unit.

### Maximal voluntary contractions

A maximal isometric voluntary contraction (MVC) of each gastrocnemius muscle was recorded to provide a metric for matching submaximal muscle contractions. Participants were in a seated position, with the knee at 90 degrees flexion and foot flat against the floor with the ankle positioned at 90 degrees to the ground. They were instructed and encouraged to contract each gastrocnemius in turn against the floor, as strongly as possible, over a 5 second period. Visual feedback of the root mean squared EMG signal within a 50 ms moving window was provided during each maximal contraction. Participants were then asked to stand unipedally on each foot in turn so that EMG amplitude could be recorded. The EMG level during unipedal stance, expressed as a percentage of the respective maximum, was then used as the target EMG level for each gastrocnemius in each experimental condition (target EMG levels were ~ 5% of MVC). A real-time column plot displayed current and target EMG levels for each trial.

### Transcranial magnetic stimulation

Participants were fitted with a tight-fitting cap and the vertex of the skull (according to the 10–20 system), indicative of the lower limb region of the primary motor cortex, was marked. Participants did not wear ear plugs. The centre of the TMS coil was placed over the vertex and active motor threshold was determined with the participant in a seated position, feet flat on the floor, knees at 90 degrees, arms resting in the lap and with the target muscle active at the reference level (~ 5% MVC). The intensity of TMS was increased from 30% of maximum stimulator output (MSO), and the TMS coil moved slightly anteriorly and posteriorly along the midline of the longitudinal fissure, until MEPs responses were observed following 3/6 stimuli (Bashir et al. 2019): according to convention, this was termed the ‘hotspot’. The TMS coil was fitted with a laser crosshair, such that once the ‘hotspot’ was determined, the position of the laser cross could be marked on the cap, indicative of the angle and position of the coil, allowing accurate positioning during experimentation. During the remainder of the experiment, TMS was applied at 120% of this active motor threshold to ensure that the stimuli elicited MEPs within the ascending limb of a typical recruitment profile, thereby avoiding high/low saturation of MEP amplitudes (Devanne et al. 1997).

### Experimental conditions

Twenty MEPs were recorded in each of four experimental conditions, in which the left and right gastrocnemii were equally activated (EMG was matched in each condition for both target muscles at the % of MVC required for unipedal stance) while providing different levels of postural support. In the four conditions, where TMS was applied, participants were asked to: (i) voluntarily activate both gastrocnemii while sitting (Sit, SI), (ii) standing bipedally (Stand, ST), (iii) voluntarily activate their right leg while standing on their left (Stand Left, SL) and (iv) voluntarily activate their left leg while standing on their right (Stand Right, SR: Fig. 1). The right gastrocnemius was considered to be providing a relatively high level of support for upright posture when the right leg was supporting stance (e.g., Stand and Stand Right conditions) and a low level of postural support when the right leg was not supporting stance (e.g., Sit and Stand Left conditions). The same definition described high levels of postural support for the left gastrocnemius when the left leg was supporting body weight during stance (see Fig. 1). TMS was delivered to the gastrocnemius hotspot in two sets of ten stimuli, with stimuli within each set separated by 6 ± 2 s so to prevent prediction of stimuli by the participant. A sham (SH) TMS condition was also performed with participants standing bipedally. In this condition, ten single TMS pulses were applied at the same interval as above with the coil oriented perpendicularly to the cortex. The sham condition acted as a control condition to account for any acoustic or vibratory effects of TMS discharge, which have been shown, in non-human primates, to activate reticular formation neurons at a latency that would be consistent with late portions of lower limb MEPs (Fisher et al. 2012). Since no measures of amplitude were taken from sham trials, 10 stimuli were applied in this condition. The researcher stood on a chair above the participant to easily place the coil in the predetermined ‘hotspot’ position.

### Data analysis

EMG data were rectified following DC offset removal using custom code in Matlab (Mathworks, MA, USA). MEP onset was visually determined by one researcher and verified by a researcher blind to the original estimate. Mean muscle activity (mV) was calculated within three time windows, and calculated from the mean of 20 MEPs in each condition per participant: a 50 ms window prior to TMS (Baseline EMG), a window from 0 to 8 ms (Early MEP: corticospinal origin) and a window from 8 to 20 ms (Late MEP: putatively corticofugal origin: Fig. 1) after MEP onset (Fisher et al. 2012; Brum et al. 2016). The longer window for the late MEP was selected in alignment with neuronal recordings from the reticular formation by Fisher et al. (2012) who observed ‘late’ firing across a range up to 25 ms after stimulation. We hoped to incorporate more of this activity into the average.

Changes in early and late MEP amplitudes across postural support conditions were assessed with a two-way repeated measures ANOVA with main factors of MEP component (early/late MEP) and Posture (4 conditions) for each gastrocnemius muscle (left and right) (SPSS: IBM Corp, NY, USA). Planned comparisons were conducted using paired *t* tests to test our four specific hypotheses. Effect sizes were expressed as eta squared for ANOVA data and proportional changes, and as Cohen’s D for paired *t* tests. Data that deviated significantly from normality were instead subjected to paired Wilcoxon signed-rank tests. To assess the relative change in MEP amplitudes between conditions, early and late MEP amplitudes during bipedal standing (high postural support) were expressed as a percentage of the corresponding MEP during sitting (low postural support). For comparisons between left and right foot standing, the gastrocnemius in the standing leg was considered to be in the high postural support condition. For the right gastrocnemius muscle, MEP amplitudes during right foot standing (high postural support) were expressed as a percentage of those measured during left foot standing (low postural support), where the right foot was not utilised. For the left gastrocnemius, MEP amplitudes during left foot standing (high postural support) were expressed as a percentage of those measured during right foot standing (low postural support). In this way, positive percentage change always represented an increased MEP in the condition of high postural support. A linear regression was also performed to test the hypothesis that changes in early and late MEPs were proportional in all transitions from low to high postural support. Input data for the regression included the percentage change in early and late portions of MEPs during transitions between: (i) Sit to Stand (data from both target muscles), (ii) Stand Left to Stand Right (data from the right gastrocnemius only) and (iii) Stand Right to Stand Left (data from the left gastrocnemius only). To determine whether tonic muscle activity prior to stimulation remained similar across conditions, a Bayesian repeated measures ANOVA with default priors was conducted using JASP (v. 0.10.2, JASP team 2019). The Bayes Factor (BF01) were reported expressing the probability of the data given the null hypothesis (H0) of no difference between conditions over the alternative (H1). Thus, values larger than 1 represent increasing favor towards the null hypothesis (Quintana and Williams 2018).

## Results

**Fig. 2:**
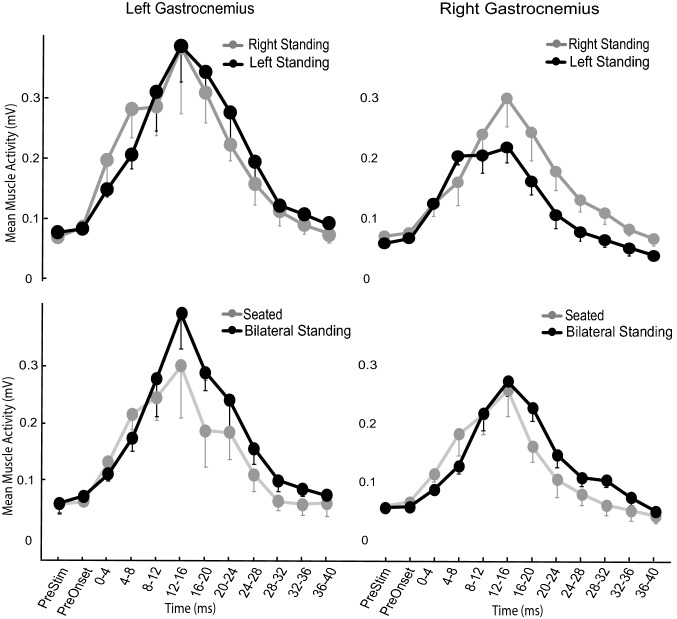
Mean muscle activity of all participants across the duration of the MEP, separated into averaged 4 ms bins. The left top and bottom panels indicate the activity in the left gastrocnemius muscle, while the right top and bottom panels indicate the activity in the right gastrocnemius across the various conditions. Error bars indicate standard deviation

**Fig. 3:**
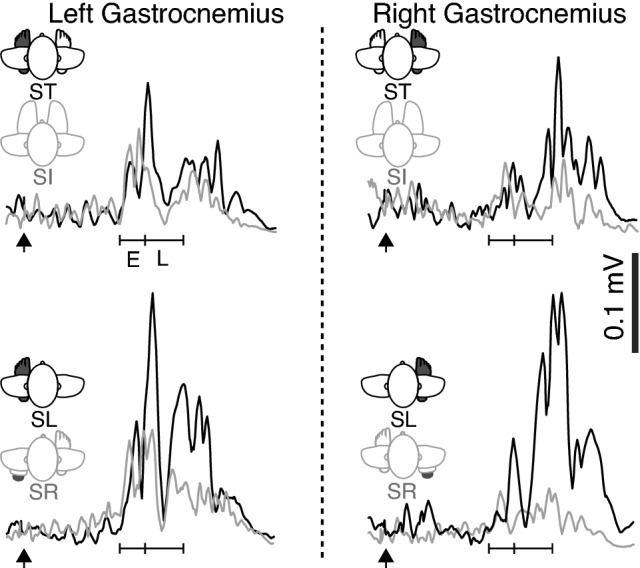
Rectified data from a single participant, each line reflecting the mean of ten trials in each condition. The left side of the figure illustrates MEPs from the left gastrocnemius, comparing Sit (SI) (grey) and Stand (ST) (black) conditions (upper panel) and Stand Left (SL) (grey) and Stand Right (black) (SR) conditions (lower panel). The right side of the figure illustrates MEPs recorded from the right gastrocnemius in the same conditions: Sit (SI) (grey) and Stand (ST) (black) conditions in the upper panel and Stand Left (SL) (grey) and Stand Right (SR) (black) conditions in the lower panel. The time of TMS application is indicated by an arrow in each panel. The time scale depicts the duration of the response classified as either early (E) or late (L) MEP, while the vertical scale is shown by the annotated thick line and indicated millivolts (mV)

**Fig. 4:**
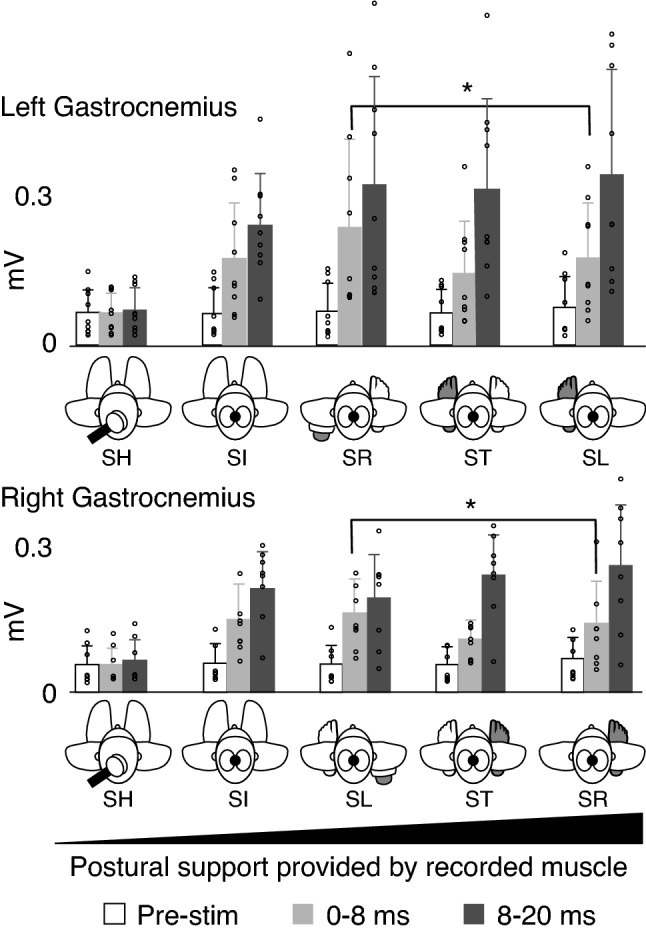
Mean (+ SD) EMG within pre-stim (white) and early (light grey) and late (dark grey) MEP windows in the left (upper panel) and right (lower panel) gastrocnemius muscles. For each muscle, the experimental conditions are ordered from the least to the most posturally demanding conditions; sham (SH), sit (SI) and then stand right (SR) or stand left (SL) depending on the leg that is not providing support, followed by stand (ST), and then stand right (SR) or stand left (SL) depending on the leg providing support. Individual data points for each condition are displayed as open circles on the respective condition. The asterix indicates significance (*p* < 0.05)

Unlike the typically observed biphasic responses of distal upper limb muscles to TMS (Edgley et al. 1990; Rothwell et al. 1990; Rossini et al. 1994), MEPs recorded in the gastrocnemius displayed a more complex pattern. Onset occurred at an average of 30.3 ± 4.4 ms following stimulation, the average active motor threshold of which was 42.9 ± 8.1% of MSO and the average with which we then stimulated was 51.7 ± 9.9%. An initial peak occurred between the first ~ 8 ms of the response (within the Early MEP, Fig. 1C) and a series of peaks occurred 8–20 ms after MEP onset (within the Late MEP period: Fig. 1C). This 0–8 ms was clearly modulated differently from the remainder of the response across the average of all participants (Fig. 2). For most participants, responses within the Late MEP period were consistently larger than those within the Early MEP period. Averaged, rectified EMG traces also suggest that Late MEPs were largest in conditions in which the target muscle functioned to provide postural support (Figs. 2 and 3A–D). An inversion between the size of the late component and the limb providing postural support was also observed (Fig. 2). No MEPs were elicited by sham TMS, indicating that neither the noise or vibration associated with coil discharge produced meaningful activation of descending motor pathways (Fig. 4).

These observations are supported by group mean data (Fig. 4). When tonic muscle activity prior to stimulation (‘Pre-stim’ data in Fig. 4) was compared, no change in tonic EMG levels across postural conditions was identified as being 7 times more likely than different EMG levels across conditions (BF01 = 7.062). Repeated measures ANOVAs (Response time X Postural support condition) performed on MEP amplitudes from each target muscle identified a significant main effect of MEP response time (early/late MEP) on amplitude in both target muscles [Right Gastroc: *F*(1,8) = 16.84, *p* = 0.003, $\eta$ ^2^ = 0.218; Left Gastroc: *F*(1,8) = 31.3, *p* < 0.001, $\eta$ ^2^ = 0.135], but no main effect of postural support condition in either muscle. In both muscles, late MEP amplitudes were significantly larger than early MEPs. There was also a significant interaction between response time (Early/Late MEP) and postural support condition on MEP amplitude in the right gastrocnemius [*F*(3,24) = 5.77, *p* = 0.004, $\eta$ ^2^ = 0.049]. Post-hoc planned comparisons performed using paired *t* tests identified significant differences in the early portions of the response rather than the late response as first anticipated. Specifically, the early component of the MEPs was found to be significantly smaller when the limb was providing postural support. In the left gastrocnemius, the amplitude of the early MEP was found to be significantly smaller in the Stand Left condition compared to the Stand Right condition [*t*(8) = 2.42, *p* = 0.042, Cohen’s D = 0.807]. In the right gastrocnemius, early MEP amplitudes were significantly different between the Sit and Stand conditions [*t*(8) = 2.39, *p* = 0.044, Cohen’s D = 0.796].

**Fig. 5:**
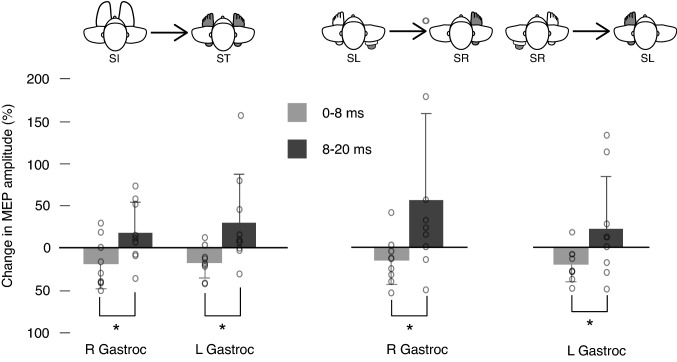
Percentage changes in early (light grey) and late (dark grey) MEP components of the motor evoked potential response from conditions of lower to greater postural demand (mean ± SD). The left panel of results shows the change from sit (SI) to stand (ST), first in the right gastroc and then in the left. The right panel of results shows the change first in the right gastroc when it goes from a position of no postural demand to single leg support, that is from stand left (SL) to stand right (SR). Then the change in the left gastroc from a stand right (SR) condition to a stand left (SL) condition is shown. Individual data points for each condition are displayed as open circles on the respective condition. The asterix indicates significance (*p* < 0.05)

Examination of the proportional changes in MEP amplitude between task conditions showed that the early and late components of the MEP were modulated differently. Figure 5 shows the proportional change in MEP amplitudes between conditions when the gastrocnemius muscle provided either low (i.e., Sit) or high (i.e., Stand) levels of postural support. When the muscle functioned to provide postural support, the late component of the MEP was proportionately larger, and the early component smaller, in comparison to the non-postural task. The proportional changes in the late and early components of the MEP between the Sit and Stand conditions were significantly different [Left gastroc: *W*(8) = 1.0, *p* = 0.012, $\eta$ ^2^ = − 0.911; Right gastroc: *W*(8) = 1.0, *p* = 0.012, $\eta$ ^2^ = − 0.911]. Similarly, significant differences were seen during unipedal stance conditions, where the late component was larger, and the early component smaller, when the muscle was providing postural support [right gastrocnemius: *W*(8) = 3.0, *p* = 0.027, $\eta$ ^2^ = − 0.822; left gastrocnemius: *W*(8) = 3.0, *p* = 0.027, $\eta$ ^2^ = − 0.822].

**Fig. 6:**
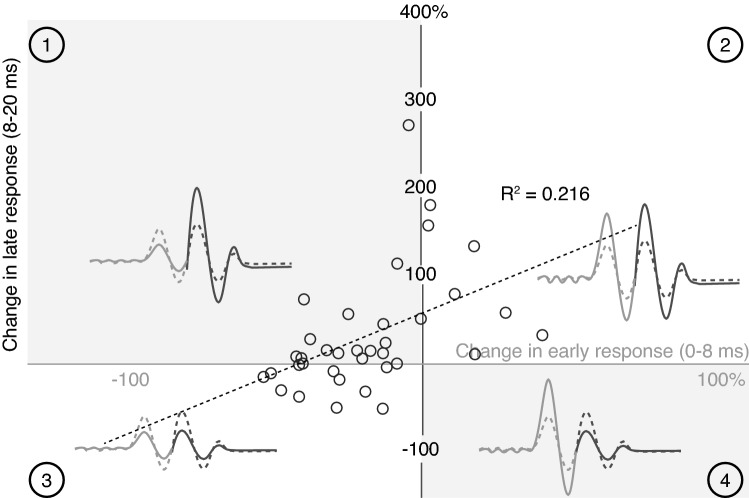
Correlation between the percentage change in the early (light grey circles) and late (dark grey circles) MEP. Each data point represents the change in MEP amplitude for a participant between either Sit and Stand or between unipedal stance conditions. As indicated by the inset waveforms, data within quadrants 1 and 4 represent changes of opposite polarity in the early and late MEP. Data within quadrants 2–3 represent changes of early and late MEP amplitude that are in the same direction. A linear regression line (dotted line) fit to the data is shown with Adj *R*^2^ value

A simple linear regression was used to test whether the change in the amplitude of the early MEP components predicted the change in late MEP component. Data from each participant and from each of the four postural transitions in Fig. 5 were included. The fitted regression model was: % Change in Late MEP = 57% + 1.349(% Change in Early MEP) (Fig. 6). This model accounted for a statistically significant amount of variability in the data (*R*^2^ = 0.216, *F*(1,34) = 9.34, *p* = 0.004).

## Discussion

In this experiment, we manipulated the amount of postural support provided by the right and left gastrocnemius to determine whether the requirement for postural support independently modulated early and late portions of MEPs induced by transcranial magnetic stimulation. Specifically, we hypothesized that increasing postural support requirements for a target lower limb muscle would preferentially increase the amplitude of late MEPs, reflecting the involvement of motor pathways descending from the brainstem in the transmission of late portions of the MEP.

While the amplitude of late MEPs was not affected by postural support requirements, our data demonstrated independent effects of posture on early and late MEPs, with preferential decreases in early MEP amplitude with increasing postural demand. Similar independent, posture-dependent changes in early/late MEP amplitude were observed in both left and right gastrocnemii. It is commonly accepted that the earliest portions of TMS-induced MEPs are due to transmission of a series of excitatory waves along the corticospinal tract (Di Lazzaro and Ziemann 2013). As such, a decrease in early MEP amplitude in conditions, where antigravity support is required from the target muscle, suggests a decrease in the involvement of cortical networks under these conditions. While we did not specifically predict a decrease in cortical involvement with increased postural demand, the differential effects of postural demand upon the amplitude of early and late MEPs supports our prediction that these responses could be independently modulated. One factor capable of causing such independent modulation would be the requirement for postural support. Our hypotheses were based upon the idea that independent modulation of early and late portions of the MEP is possible due to different descending pathways being preferentially responsible for transmitting each MEP component. It has been demonstrated in non-human primates, for example, that TMS can readily activate neurons within the reticular formation with TMS intensities as low as 40% of MSO (Fisher et al. 2012). It has also been suggested that late-arriving MEPs in a proximal muscle (pectoralis major) ipsilateral to the stimulated primary motor cortex potentially traverse a polysynaptic cortico-reticulospinal pathway (MacKinnon et al. 2004). Any cortico-reticulospinal or cortico-vestibulospinal transmission is likely to be subject to changes in excitability caused by the requirement for postural support, since motor tracts descending from the brainstem to ventromedial locations in the spinal grey matter (primarily vestibulospinal and reticulospinal tracts) have been shown to play a crucial role in anti-gravity postural support (Lawrence and Kuypers 1968b). They respond powerfully in cats, to both predictable (Schepens and Drew 2003, 2004) and unpredictable (Stapley and Drew 2009) disturbances to standing posture. On this basis, our interpretation is that the independent modulation of early and late MEPs in this study reflects the strong influence of a cortico-brainstem pathway on late MEPs, contrasting the almost exclusive transmission of early MEPs along corticospinal fibres.

In contrast to our hypothesis, the independence of early and late MEPs was demonstrated as a decrease in amplitude of early MEPs, rather than an increase in late MEPs. The decrease in early MEP amplitude contrasts with previous demonstrations of increases (Tokuno et al. 2009; Baudry et al. 2015) or no change (Soto et al. 2006) in total MEP amplitude with transitions from seated to standing postures. Since none of these studies examined the possibility of differential changes in early and late portions of the TMS-induced MEP, any decrease in early MEP amplitude is likely to have been masked by larger potentials generated during the late MEP. Since the portion of the MEP that we have defined as the late MEP is typically of greater amplitude than the early MEP in triceps surae muscles (see Fig. 1), it is likely that these experiments measured amplitude between EMG peaks that occurred within the late MEP. The reduction in early MEP amplitude with increases in postural support requirements in both legs in this study strongly suggests that the excitability of direct corticospinal projections is downregulated when anti-gravity support is raised. This may be due to either a reduced involvement of the cortex in anti-gravity support, or perhaps to an increased reliance on faster subcortical reflex responses to compensate for balance perturbations in a less stable posture. Evidence of reductions in upper limb function with increased requirements for anti-gravity support, and vice versa, has been used to suggest that anti-gravity support is preferentially provided by brainstem networks that are less capable than the cortex of dexterous movement coordination (Ellis et al. 2007; Beer et al. 2007). Animal studies have also demonstrated robust firing of reticular formation cells in response to balance perturbations, where 44/48 reticular neuronal recordings in a cat were activated upon perturbation (Stapley and Drew 2009). Both antigravity support requirements and an increased likelihood of balance perturbation may, therefore, proportionally decrease the role of the cortical motor commands.

A number of factors may explain the lack of late MEP modulation with changes in stability requirements. The first is that the balance tasks in this study were not sufficiently destabilising to induce significant modulation of the excitability of pathways contributing to late MEPs. This is certainly feasible, as the tasks were very familiar to the participants and not designed to be overly challenging, since excess sway during the standing trials would have been a confounding factor in our analyses. However, given that we observed significant changes in early MEP amplitude across postural conditions, it seems that the participants did alter the state of their descending tracts to compensate for changes in stability, the changes in excitability simply being more detectable in the early MEP. Changes in the early response are likely to have been more detectable due to the greater variability observed in the amplitude of late MEPs and magnitude of changes in that response across conditions (Fig. 6). Evidence of iMEPs being delayed by between 1.2 and 9 ms in upper limb muscles (MacKinnon et al. 2004) also suggest that the timing of cortio-brainstem contributions to MEPs is likely to have varied considerably between participants in the current study.

Perhaps unsurprisingly given the variation in human movement repertoires, our study also provided some evidence of individual variation in control strategies used to deal with changes in postural challenge. For example, one participant in this study was a trained ballet dancer and we observed that their early MEPs were modulated robustly across postural conditions to combat postural challenge, while their late component MEPs varied less. We suspect that this pattern of results may be the result of their years of training to couple precise voluntary movements with body stabilisation. An anatomical corollary of this idea has been demonstrated in a study using MRI techniques to demonstrate more diffusivity in tracts descending from the primary motor cortex in dancers than control participants or musicians, perhaps indicative of more collateral connections from pyramidal output neurons in dancers (Giacosa et al. 2019). While we have no way of knowing the effect that greater collateral connectivity had on the results for the ballet dancer in our study, it is likely that individuals with this type of training history will have different neurophysiological strategies for dealing with instability than people without similar training. It is also plausible that individuals with more diffuse descending motor connectivity would have greater capacity to coordinate their balance responses above the level of the brainstem. It appears that dancers are at least likely to regulate stability above the level of spinal segmental reflexes, since there is evidence that not only are H-reflexes in their plantarflexor muscles smaller in general than untrained individuals (Nielsen et al. 1993), but they demonstrate smaller gain changes when moving from stable to unstable postures (Mynark and Koceja 1997).

Importantly, the changes in MEP amplitude observed in our data with altered postural demand were not accompanied by any difference in tonic muscle activity (Fig. 4). This makes it unlikely that reductions in early MEP amplitude were due to changes in the excitability of the motoneuron pool. To maintain the necessary muscle activation in non-weight-bearing muscles, subjects were asked to voluntarily contract the target muscle. The change between, for example, Sit and Stand conditions was, therefore, not simply a matter of increasing the need for postural support, but also involved potentially decreasing voluntary muscle activation. Since weak voluntary muscle contraction is strongly and causally associated with cortical activity (Maier et al. 1993), this may also explain the decrease in early MEP amplitude between voluntary and postural conditions.

On the basis that early and late MEPs were transmitted by largely independent pathways, we had hypothesized that no relationship would exist between changes in the two MEP components. We did, however, identify a linear relationship between posture-induced changes in early and late MEP amplitude. Although a relationship exists, the relationship describes a system in which independent modulation is a defining characteristic, since it predicts that a 10% increase in early MEP amplitude due to a change in posture will result in a ~ 110% increase in the late MEP. Looking at the data itself (Fig. 6), a consistent rule appears to be that an increase in postural demand results in an increase in the importance of the late MEP (and hence the excitability of the pathway(s) transmitting the late MEP). Regardless of whether there is a decrease in early MEP amplitude or an increase in the late MEP amplitude, the result is a proportional increase of the late MEP in relation to the early MEP. More than half (19/36) of the data points in Fig. 6 reflect changes of opposing polarity in early and late MEP amplitude and there were no circumstances in which increased postural support was accompanied by an increased early MEP and decreased late MEP.

Several limitations in study design may explain deviations from some of our hypotheses. In retrospect, our hypotheses may have failed to consider all possible strategies for increasing the relative excitability of non-corticomotoneuronal pathways. If, as is likely, the corticomotoneuronal pathway continues to contribute to the MEP beyond 8 ms, observations of no change in the amplitude of late MEPs at the same time as decreases in the early MEP suggests a reduction in corticomotoneuronal excitability generally and a compensatory increase in the excitability of pathways contributing to the late MEP. Our data suggest that some individuals in this study used this strategy when encountering low stability conditions. Given the high variability of MEP amplitudes observed, particularly for late MEPs, a study with larger participant numbers may be able to detect an increase in late MEP amplitude with increased stabilization requirements. Higher variability of the late MEP would be consistent with transmission of this response along a polysynaptic pathway, so exploration of this variability would be a useful addition to future studies. The determination of MEP onset in studies of this nature is particularly important, and algorithms tested for this purpose have so far proved insufficiently accurate. For that reason, onsets were visually detected in mean MEPs and compared between multiple investigators in this study but the development of accurate algorithms for this purpose in future studies would remove subjectivity from this process. Despite these limitations, the results of this study provide useful insights into changes in the excitability of parallel descending motor pathways caused by changes in the level of postural stability.

Our data provide clear evidence that changes in anti-gravity postural support requirements are capable of inducing independent modulation of early and late TMS-induced MEPs. This result supports the idea that early (0–8 ms) and late (8–20 ms) components of the TMS response are transmitted along largely independent descending pathways, with the corticospinal tract transmitting the early MEP and a corticofugal pathway the most likely candidate, in our opinion, for transmission of the late portion of the response.

## Data Availability

Upon publication data will be available in the Open Science Framework.

## Code availability

Upon publication data analysis code will be available in the Open Science Framework.
